# Mini-review on the novel synthesis and potential applications of carbazole and its derivatives

**DOI:** 10.1080/15685551.2023.2194174

**Published:** 2023-03-29

**Authors:** Zhichao Xu, Di Wu, Cong Fang, Yuanzhe Li

**Affiliations:** a School of Information and Business Management, Dalian Neusoft University of Information, Dalian, China; b School of Design, The Hong Kong Polytechnic University, HongKong SAR, HongKong; c School of Materials Science & Engineering, Nanyang Technological University, Singapore

**Keywords:** Carbazole, Microporous organic polymers (MOPs), synthesis and characterization, potential applications

## Abstract

Microporous organic polymers (MOPs) are a new type of porous materials, which have advantages of synthetic diversity, chemical and physical stability, microporous size controllability, etc. MOPs indicate broad applications in various fields such as heterogeneous catalysis, gas adsorption, separation, and storage. In recent years, MOPs have attracted an enormous attention in greenhouse gas capture due to their great potential in physisorptive gas storage. Carbazole and its derivatives have been studied extensively as Metal-Organic Polyhedra (MOPs) building blocks due to their unique structural features and versatile functionalization possibilities. This paper systematically reviews the synthesis, characterization and application of carbazole-based polymers, and relationship of structures and properties of these polymers. The application of the polymers in carbon dioxide (CO_2_) capture field is analysed taking advantage of their adjustable microporous structure and electron rich properties. This review also provides novel insights regarding functional polymer materials that have high ability of greenhouse gas capture and absorbing selectivity will be obtained by reasonable molecular design and efficient synthesis.

## 1. Introduction

Carbazole is a nitrogen-containing heterocyclic compound, which is an intermediate of many fine chemicals and can be used to make plastics, pesticides, insecticides, pharmaceuticals and new polymer materials [1]. The introduction of various substituent groups or functional groups at specific positions of carbazole molecules can be used to chemically modify the carbazole derivatives to obtain a variety of novel structures [2]. With the growing demand for research and production, the demand for carbazole and its analogues has been increasing year by year. Although the compounds synthesized by traditional methods are more economical, the products are single and cannot meet the diversity needs. Therefore, it is of considerable importance to study the synthesis method of carbazole and make it easy for industrial production. The organic electroluminescence technology has its outstanding advantages over other display technologies, such as low power consumption, easy bending, fast response time, wide viewing angle, and large-area display. In the realization of color flat panel display with a full range of light-emitting colors such technology can be made with a variety of existing standards, technology compatible with low-cost light emitters, which shows its strong vitality [1,3]. It is these potential advantages that the research of organic electroluminescence technology has attracted great interest from many researchers at home and abroad. In contrast, certain conjugated polymers containing carbazole groups have superior optical properties compared to conventional conjugated polymers. From the structural point of view, the carbazole electrophilic Nitrogen (N atom) absorbs electrons from the double bond through the induction effect. Moreover, carbazole groups have superior optical properties compared to conventional conjugated polymers due to their unique molecular structure. The carbazole moiety possesses a planar π-conjugated system with high rigidity and electron delocalization, which allows for efficient light absorption and emission [1,2]. Additionally, the presence of nitrogen atoms in the carbazole ring enables the formation of charge transfer complexes, resulting in red-shifted absorption and emission spectra compared to conventional conjugated polymers. These properties make carbazole-based materials attractive candidates for optoelectronic applications, such as organic light-emitting diodes (OLEDs), photovoltaics, and sensors [4]. This kind of hole transport material reduces the crystallization of small molecule materials and improves the device lifetime. Moreover, it increases the chance of electron 2-hole complexation and improves the luminescence efficiency of the device [5]. In recent years, some scholars have started to study carbazole-based organic microporous polymers, because the hydrogen at the 3 and 6 positions on the carbazole is very active and can be easily condensed after joining to the polymer, thus obtaining hyperbranched microporous polymers. The microporous size of these polymers is generally below 2 nm, and they can adsorb a certain amount of CO_2_ at room temperature [6]. Meanwhile, due to the electron-absorbing effect of N on carbazole, some physical adsorption can be formed between these microporous polymers and CO_2_, which can adsorb greenhouse gases more effectively. It may open up a new field of application of carbazole-based polymers to explore the ways to solve global warming [6,7]. Given the promising optical and electronic properties of carbazole and its derivatives, there is a growing interest in designing innovative synthetic strategies to further enhance their performance in various optoelectronic applications. Therefore, the development of new and efficient synthetic methods for carbazole derivatives holds great promise in advancing the field of materials science and benefiting the broader community. By improving the performance of optoelectronic devices through the design and synthesis of novel carbazole derivatives, we can pave the way for a more sustainable future and contribute to the development of next-generation technologies.

## 2. The synthesis method of carbazole

### 2.1 Traditional methods of carbazole synthesis

**Figure 1.**
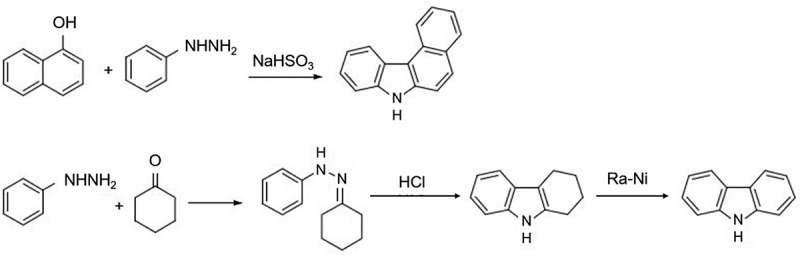
Aryl hydrazine by co-thermal reaction with naphthol in the presence of sodium bisulfite and cyclized under acidic conditions to produce tetrahydrocarbazole and catalytically dehydrogenated to produce carbazole.

The traditional synthesis methods of carbazole mainly include Graebe-Ullmann reaction, Bucherer carbazole synthesis and Borsche-Drechsel cyclization reaction. Graebe and Ulmann reported the Graebe-Ulmann reaction in 1896, in which carbazole was obtained from aminodiphenylamine by diazotization and heating off the nitrogen, as shown in Figure 1 [1]. Ullmann synthesized several carbazole compounds by this method, but other researchers found that the presence of unsaturated groups adversely affected the reaction. Preston, Tucker and Cameron [2] obtained nitrocarbazole (trace), acetylcarbazole (22%) and cyanocarbazole (34%) by this method for the first time, but the yields were not satisfactory. Bucherer carbazole synthesis was reported by Bucherer in 1904, where benzocarbazole was synthesized from aryl hydrazine by co-thermal reaction with naphthol in the presence of sodium bisulfite [3]. The Borsche-Drechsel cyclization reaction was reported by Drechsel and Borsche in 1868 and 1904, respectively [1,3], in which phenylhydrazine was condensed with cyclohexanone to form hydrazones, which were cyclized under acidic conditions to produce tetrahydrocarbazole, which was then catalytically dehydrogenated to produce carbazole [4]. Thanks to the development of catalytic dehydrogenation methods, Borsche’s method has become a simple, mild, low-cost, and high-yield route for carbazole synthesis, which has high industrial production value [3,4].

**Figure 2.**
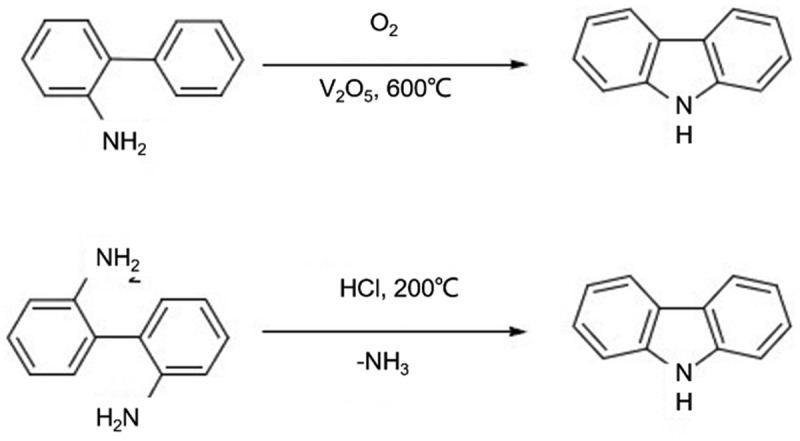
Oxidative dehydrogenation of 2-aminobiphenyl or deamination of 2,2’-diaminobipheny for the preparation of carbazole.

In addition, carbazole is also often prepared industrially by oxidative dehydrogenation of 2-aminobiphenyl or deamination of 2,2’-diaminobiphenyl [5], as shown in Figure 2. The reaction yield of the synthetic scheme for the preparation of carbazole through oxidative dehydrogenation of 2-aminobiphenyl or deamination of 2,2’-diaminobiphenyl can vary depending on several factors, including the reaction conditions, the purity of starting materials, and the choice of catalyst. However, in general, these synthetic methods have been reported to provide moderate to high yields of carbazole [4,5]. For example, the oxidative dehydrogenation of 2-aminobiphenyl using various metal catalysts such as CuCl, Cu(OAc)_2_, and Cu(OAc)_2_/TEMPO has been reported to provide yields ranging from 40% to 94% under optimized conditions. Similarly, the deamination of 2,2’-diaminobiphenyl using various oxidants such as CuCl_2_, MnO_2_, and PbO_2_ has been reported to provide yields ranging from 55% to 98% [5,6].

### 2.2 Novel methods of carbazole synthesis

In the last decade of the 20th century, a large number of novel noble metals and transition metal catalysts were applied in the synthesis of carbazole and its derivatives, which greatly enriched the synthetic tools and laid the foundation for the synthesis of more complex carbazole derivatives. Instead of simply synthesizing carbazole, thousands of carbazole derivatives have been synthesized by new methods and are widely used in the pharmaceutical and dye industries. In this paper, the synthesis methods of carbazole and its derivatives are classified into four categories according to the different intermediates: biphenyl, diphenylamine, indole, and others.

#### 2.2.1 Biphenyl as the intermediate

**Figure 3.**
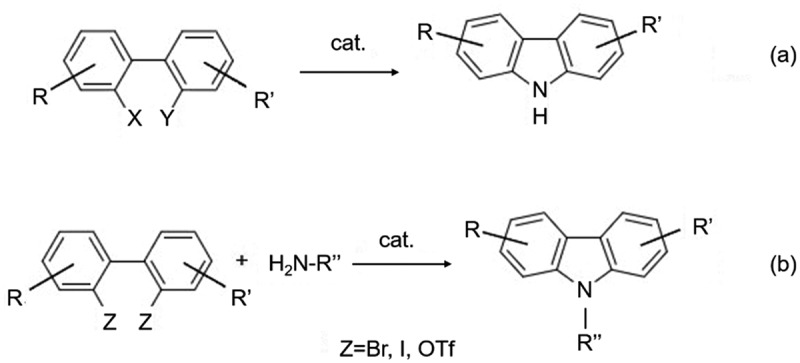
(a) Suzuki-Miyaura coupling reaction to synthesize biphenyl intermediates with different substituents and (b) cyclization using different nitrogen-containing groups and catalysts.

The synthetic method using biphenyl as an intermediate is based on the discovery of the Suzuki-Miyaura coupling reaction. The basic synthetic strategy of this method is to use the Suzuki-Miyaura coupling reaction to synthesize biphenyl intermediates with different substituents firstly, and then to obtain the targets by cyclization using different nitrogen-containing groups and the corresponding catalysts (Figure 3). Compared with Ullmann’s method, the reaction temperature of Suzuki-Miyaura coupling usually is less than 100°C. Moreover, the reaction can be carried out in a mixture of water, alcohol, and ether, which has significant advantages in terms of reaction conditions, yield, and application. For example, 4-hydroxycarbazole is an important intermediate of carbazole cardiac (for hypertension).

When the synthesis process uses a large excessive amount of copper powder as catalyst and the reaction occurs at 185℃, the yield of biphenyl preparation is only 50%-60%. However, when Suzuki-Miyaura coupling is catalyzed by Pd, the yield can be increased to 95%-100%, the catalyst dosage can be reduced to 5 mol%, and the temperature can be lowered to about 90°C, which can save a lot of energy, reduce environmental pollution, and improve efficiency.

**Figure 4.**
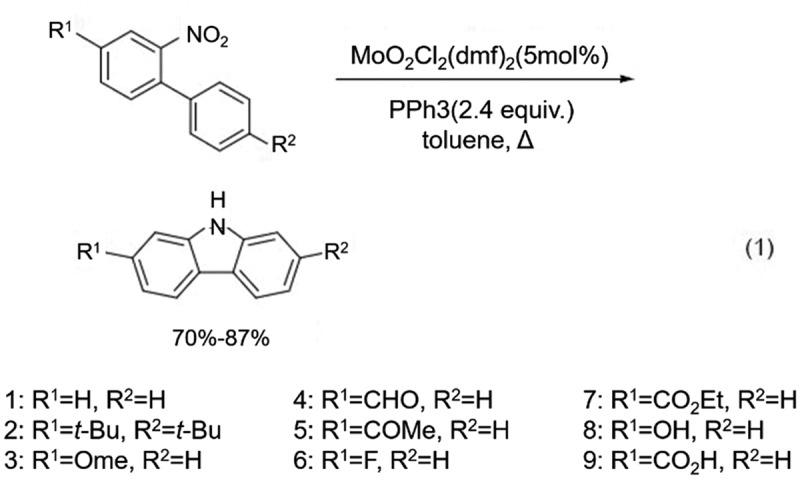
Synthesis of carbazoles and carbazole derivatives using disubstituted nitrobiphenyls in presence of MoO_2_Cl_2_(dmf)_2_ and PPh3.

**Figure 5.**
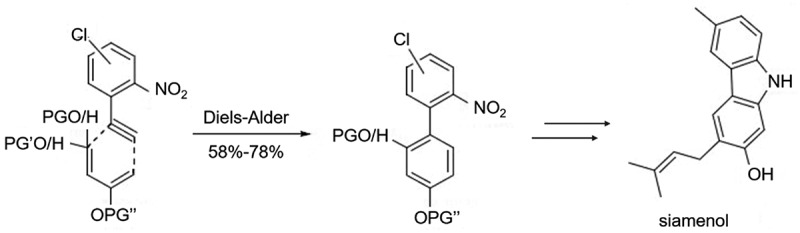
Siamenol synthesized process by cyclization of PPh_3_/dichlorobenzene system using nitrobiphenyl as the key intermediate.

In the cyclization step, the main ring-forming groups that can be utilized are NO_2_, N_3_, NHR, etc. When X and Y are halogens or trifluoromethanesulfonate (OTf), additional amino compounds are required as the nitrogen source for the carbazole. Among the various cyclization reactions, nitro is the most widely used as a cyclization group. Arnáiz group [6] synthesized a series of carbazoles and carbazole derivatives 1 ~ 9 in 70% − 87% yield (Figure 4) by using various disubstituted nitrobiphenyls as raw materials with MoO_2_Cl_2_(dmf)_2_ as catalyst and in the presence of PPh3. PPh3 is likely used as a ligand to coordinate with the MoO_2_Cl_2_(dmf)_2_ catalyst, which enhances its ability to activate the nitrobiphenyl starting materials. The mechanism of the catalytic reaction likely involves the coordination of the MoO_2_Cl_2_(dmf)_2_ catalyst with PPh3 to form an active species that can activate the nitrobiphenyls through a redox process. In 2007, Carter et al. [7] reported that multi-substituted nitrobiphenyl intermediates could also be obtained using the Diels-Alder reaction with yields of 58%-78%, showing better stability than the Suzuki coupling method (22%-98%). A series of carbazole derivatives, such as Siamenol, could also be synthesized by cyclization of PPh_3_/dichlorobenzene system using nitrobiphenyl as the key intermediate (Figure 5).

**Figure 6.**
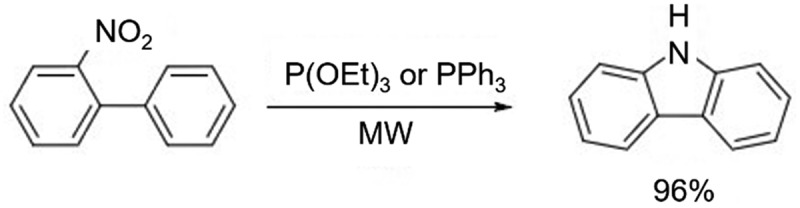
Carbazole synthesis in the presence of P(OEt)_3_ or PPh_3_ with a microwave power.

Normally, the cyclization reaction of nitrobiphenyl requires high temperature and long reaction time (5–12 h) to complete the reaction. In 2009, Creencia et al. [8] reported a method for the microwave-assisted synthesis of carbazole from nitrobiphenyl in the presence of P(OEt)_3_ or PPh_3_ with a microwave power of 200 W. The reaction was completed in only 2 min with a high yield of 96% (Figure 6).

#### 2.2.2 Azide groups as intermediates

**Figure 7.**
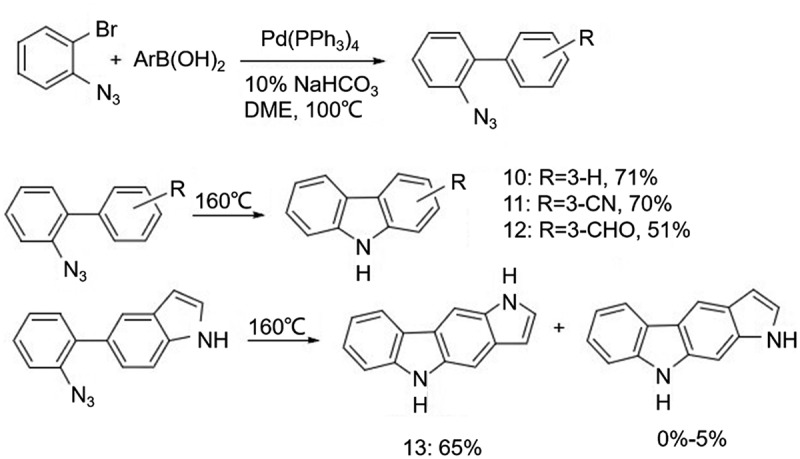
Carbazole synthesis obtained by releasing nitrogen gas through heating.

**Figure 8.**
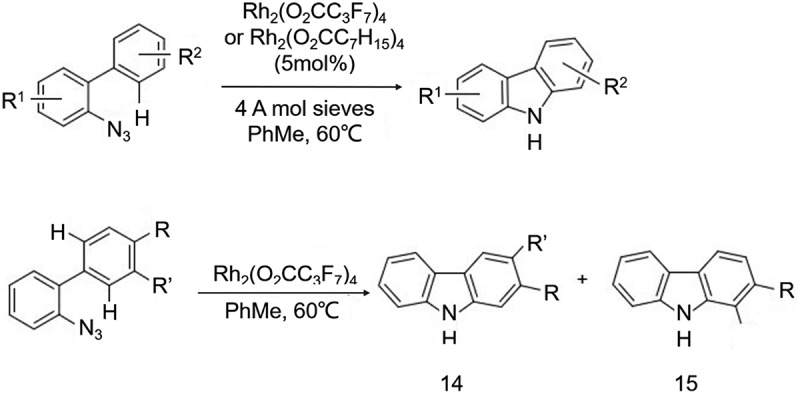
Carbazole derivatives synthesis using azidobiphenyl under the conditions of Rh_2_(O_2_CC_3_F_7_)_4_.

Use of azide group as a cyclic group is also an effective method for synthesizing carbazole. In 2007, Sapi et al. [9] obtained azidobiphenyl by Suzuki-Miyaura coupling reaction, and then carbazole was obtained by releasing nitrogen gas through heating (Figure 7). The experimental results showed that there was a great variation in the yield when the substituents on the azobiphenyl were different, fluctuating from 51% to 71%. It is found that the product has good selectivity when the biphenyl intermediate is benzoindole at one end, indicating that the electrical characteristics of the substituent have an important influence on the ring-combination site. In 2009, Driver et al. [10] synthesized a total of 23 carbazole derivatives using azidobiphenyl under the conditions of Rh_2_(O_2_CC_3_F_7_)_4_ (5 mol%), 4 ÅMS (100 wt%), PhMe, 60°C and Rh_2_(O_2_CC_7_H_15_)_4_ (5 mol%), 4 ÅMS (100 wt%), (CH_2_Cl)_2_, 60°C, respectively. This method showed high yields of 65% to 98% (Figure 8), suggesting a clear advantage of rhodium-catalyzed reactions. This study also discussed the regioselectivity (14:15) during cyclization. 14:15 > 95:5 when R’ is chlorine or trifluoromethyl, 89:11 when R is fluorine, but only 66:34 when R is methyl and almost no selectivity when it is methoxy, which further complements the theory of Sapi et al. on the relationship between substituents and cyclic sites.

**Figure 9.**
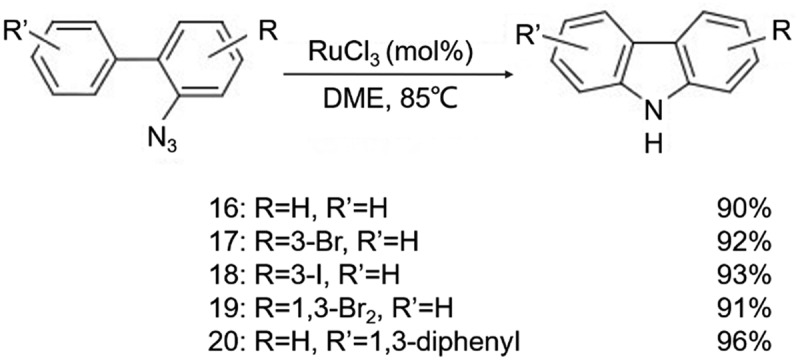
Ruthenium complexes series preparation [e.g., RuCl_2_-(PPh_3_)_3_, RuCl_2_(DMSO)_4_, RuCl_3_, RuO_2_, (NH_4_)_2_RuCl_6_] for the C-H amination reactions of organic azide compounds.

**Figure 10.**
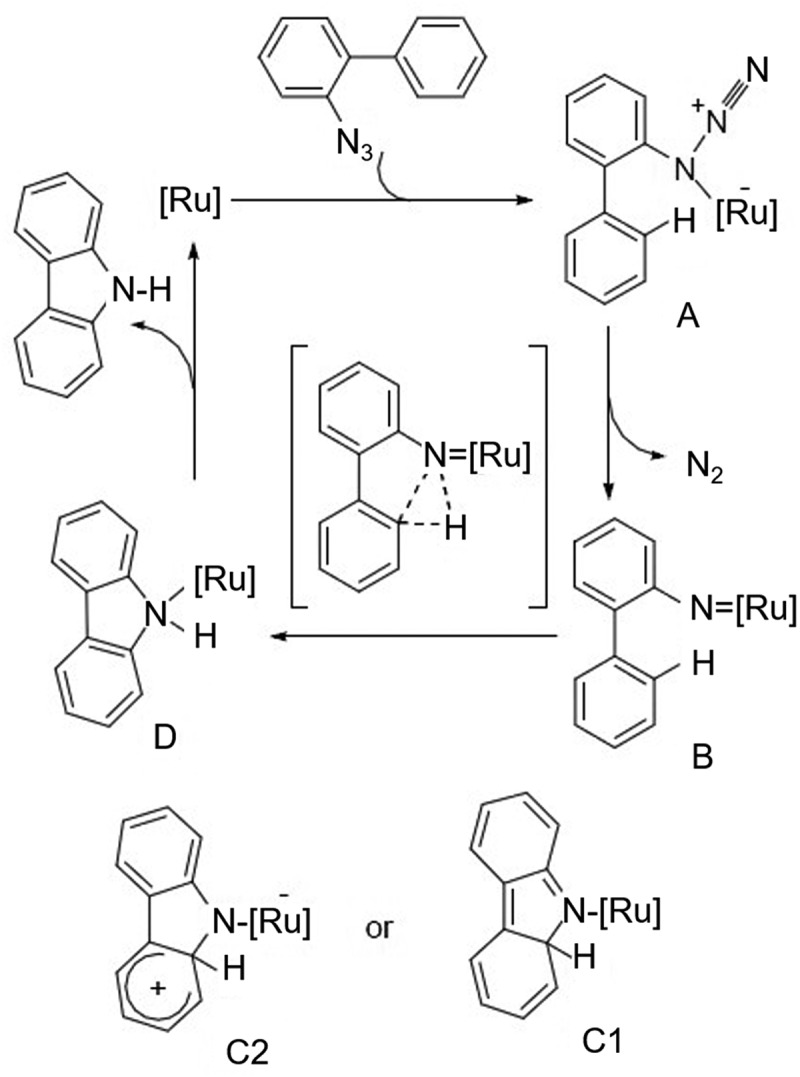
Aryl substitution on the aryl ring opposite to the azide group.

In 2009, Jia et al. [11] studied the catalytic activity of a series of ruthenium complexes [e.g., RuCl_2_-(PPh_3_)_3_, RuCl_2_(DMSO)_4_, RuCl_3_, RuO_2_, (NH_4_)_2_RuCl_6_] for the C-H amination reactions of organic azide compounds (Figure 9). RuCl_3_ showed the best catalytic activity and the reaction proceeded favorably when there was aryl substitution on the aryl ring opposite to the azide group, with a reaction time of only 1.5 h and a yield of up to 96%, as shown in Figure 10. The authors speculate that during the reaction, the organic azide compound firstly reacts with the ruthenium catalyst to form a compound, and forms intermediate B after releasing nitrogen gas, and finally may be converted into intermediate D through two rearrangement pathways. Intermediate D is a ruthenium complex with carbazole as the ligand, and the target product carbazole is finally obtained after decomposition.

**Figure 11.**
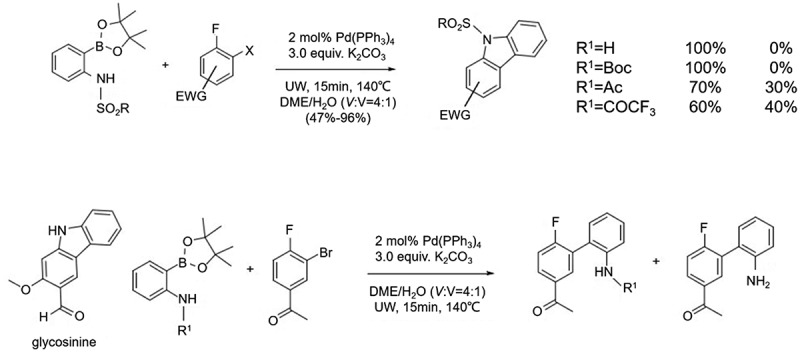
Direct synthesis of carbazoles from N-substituted aminophenylboronic ethers and o-dihalogenated benzenes.

Recently, it was reported that aminobiphenyl can also be used for the preparation of carbazole, especially for the synthesis of *N*-substituted asymmetric carbazole. Recently, it has been reported that aminobiphenyl can also be used for the preparation of carbazole, especially for the synthesis of N-substituted asymmetric carbazole. In 2007, Jean et al. [12] reported the direct synthesis of carbazoles from N-substituted aminophenylboronic ethers and o-dihalogenated benzenes by a one-pot method via Suzuki-Miyaura coupling reaction and intramolecular SNAr reaction (Figure 11). The N-substituted groups included Boc, Ms, Ts, Ac, and COCF3. The experimental results showed that only the sulfonyl groups (Ms and Ts) were substituted and all the products were carbazoles, while the other substituents only obtained the corresponding biphenyls, in which Ac and COCF3 were substituted and about 30%-40% of the biphenyls obtained were stripped of Ac and COCF_3_, suggesting that the practical use of this method is limited.

In 2008, Gaunt [13] and Buchwald’s group [14] synthesized a series of asymmetric carbazole derivatives in high yields by the amination reaction of C-H to form C-N bonds using Pd(OAc)_2_ as catalyst, respectively. The substituents in the synthesized carbazole derivatives were not only concentrated on one of the benzene rings, but also distributed on both benzene rings of the carbazole, indicating that the method can be widely used for the synthesis of different multi-substituted carbazole derivatives. Pd(OAc)_2_ can activate the aryl halide and boronic acid through oxidative addition and transmetallation steps, leading to the formation of an active Pd species that can promote the desired C-N and C-C bond-forming reactions [13,14].

**Figure 12.**
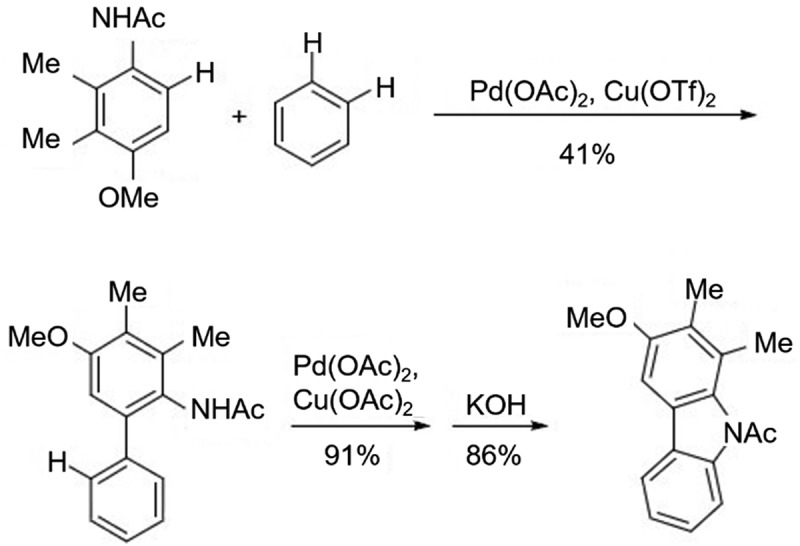
Synthesis of carbazoles using 2,3-dimethyl-4-methoxyacetanilide and benzene.

**Figure 13.**
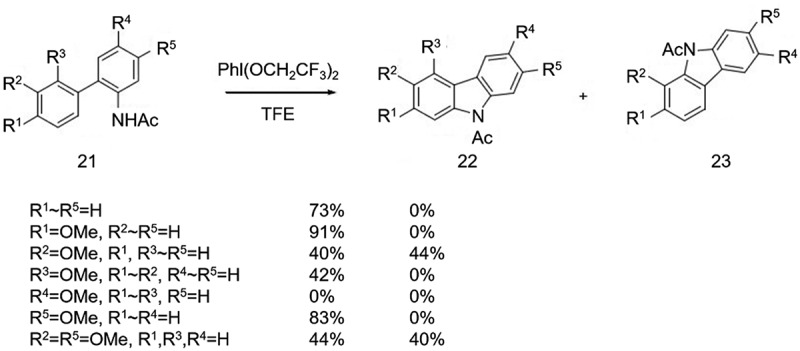
Generation of by-products when the biphenyl intermediates with different substituents.

In 2008, Shi et al. [15] developed a new coupling approach for the synthesis of carbazoles using 2,3-dimethyl-4-methoxyacetanilide and benzene as raw materials and activating the C-H bond with Pd(OAc)_2_ and Cu(OTf)_2_ as catalysts, followed by Pd(OAc)_2_ and Cu(OAc)_2_ as catalysts to synthesize carbazole (Figure 12). Although the yield in the first step was not that high, this is a novel coupling approach, which has the advantage of eliminating the need to introduce boronic acid and halogen substituents at specific positions of the feedstock compared with the Suzuki-Miyaura coupling reaction. But the regioselectivity problem of this method in the cyclization remains to be explored. Nishiyama et al. [16] studied systematically the application of oxidative cyclization reaction induced by the hypervalent iodine compound PhI(OCH_2_-CF_3_)_2_ in the synthesis of carbazole. It was found that the new high-valent iodide PhI(OCH_2_-CF_3_)_2_ was efficient in obtaining carbazole products under milder conditions compared to the productized Phenyliodine(III)bis(trifluoroacetate)[PIFA]. While PIFA had lower yields at low temperatures and was associated with by-products when the biphenyl intermediates with different substituents (Figure 13).

Chang et al. [17] recently reported the synthesis of Cu-catalyzed intramolecular oxidative coupling to form C-N bonds, which can be widely used for carbazole synthesis. The raw materials for the synthesis was benzenesulfonamide-substituted biphenyl, and the reaction was catalyzed by Cu(OTf)_2_ in the presence of trifluoroacetic acid to obtain carbazole in more than 90% yield in 10 min. This method is undoubtedly more advantageous than the aforementioned methods in terms of operation and yield, but whether it can be extended for the synthesis of mono-/multi-substituted carbazole derivatives has not been explored in this report.

**Figure 14.**
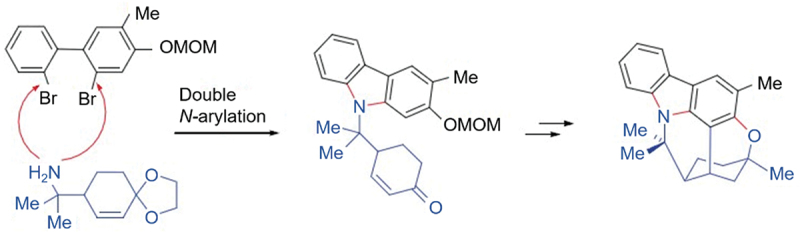
Dihalogen-substituted biphenyls and primary amines as raw materials for the total synthesis of (±)-Murrayazoline.

Carbazole can also be synthesized from dihalogen or hydroxyl substituted biphenyl. In 2008, Chida’s group [18] applied dihalogen-substituted biphenyls and primary amines as raw materials for the total synthesis of (±)-Murrayazoline by intramolecular Friedel-Crafts Michael addition reaction and Pd-catalyzed C-O coupling, one of the key steps of which was the construction of the carbazole ring (Figure 14).

Carbazole has attracted increasing interest from researchers due to its unique structure and physicochemical properties, especially in recent years, new mono- and multi-substituted carbazole derivatives have been found to have good antitumor and anticonvulsant activities, showing promising applications. As a result, the demand for carbazole and its derivatives is increasing. Although conventional carbazole preparation methods such as Borsche synthesis have the advantages of easy operation and low-cost, they are only capable of synthesizing individual simple carbazole derivatives, but not complex carbazole derivatives, especially those with specific sites and specific substituent groups. Therefore, it is useful to develop novel methods for the synthesis of carbazole and its derivatives. The development of catalytic science and the application of noble metal and transition metal complex catalysts have greatly contributed to the development of methods for the synthesis of carbazole and its derivatives. In particular, Suzuki coupling reaction, Domino electrocyclization reaction and intramolecular oxidative coupling reaction have made the synthesis of complex carbazole derivatives increasingly easy, and the large-scale preparation of complex carbazole derivatives has gradually changed from theory to reality. With the continuous research, the scope of application of carbazole and its derivatives will be broadened and various new synthetic methods will be emerged. The research on the synthesis of carbazole and its derivatives is far from over and still needs to be further enriched and developed.

## 3. Analysis of the research prospects of carbazole

The novel microporous organic polymer materials presented in this paper can be useful in non-homogeneous catalysis, adsorption, separation and gas storage [19,20]. Conventional microporous solids include inorganic crystalline skeletal structures (e.g., molecular sieves and their related structures) and amorphous network structures (e.g., porous silica and activated carbon). In recent decades, the assembly of microporous organic polymers by organic building blocks has shown great superiority, generally with high specific surface areas (SSAs) and polymer molecular chains mainly composed of low density elements C, N, O, B, etc. Compared to carbon materials such as activated carbon (AC), graphite and metal-organic coordination polymers (MOFs), they can be functionalized to a large extent by changing the functional groups of organic molecules and using different synthetic means to modulate the microporous polymers [21]. In addition, most organic polymers are usually very stable to air, ambient humidity or more demanding environments [22]. Thus, microporous organic polymer materials are gradually developing as a new and highly promising material for gas storage, especially for hydrogen storage. The main microporous organic polymers with these characteristics are Hypercross-linked Polymers (HCPs), Polymers of Intrinsic Microporosity (PIMs), Conjugated Microporous Polymers (CMPs), and Covalent-Organic Frameworks (COFs). Microporous Polymers (CMPs), and Covalent-Organic Frameworks (COFs). Among them, PIMs have unique requirements in the structure of constituent monomers; CMPs have great prospects for organic optoelectronic devices.

### 3.1 Hypercross-linked polymers

HCPs are constructed by hyperbranched polymer chains to prevent the dense packing between the chains, enabling the construction of microporous structures. It was originally developed by Tsyurupa et al. [23] and applied to column chromatography, where it was found to be able to separate wastes from wastewater as well as adsorb organic vapors very well. Firstly, the solvent for crosslinking should be as thermodynamically compatible as possible with the precursor; secondly, the crosslinking reaction should occur as fast as possible; and finally the reagents and solvents chosen for the reaction should not interact chemically [24]. This type of microporous polymers are amorphous polymers with high SSAs up to 2090 m^2^g^−1^ at present [25], but few examples have been studied. It is mainly synthesized by two methods, one is to obtain precursors by cross-linking chloromethylstyrene (VBC) as monomer with cross-linking agent divinylbenzene (DVB) and then obtain polymer networks with super-cross-linked structures Friedel-Crafts reaction [25,26]. The advantage of this synthetic method is that different SSAs can be obtained by controlling the ratio of crosslinker to monomer [26,27]. Another synthetic method is the one-step synthesis of monomers containing two chloromethyl groups on the aromatic ring (or a mixture of several monomers of similar structure) by the Friedel-Crafts reaction [28]. The monomers used in this method are mainly p-dichlorodimethylbenzene (p-DCX), 4,4′-dichloromethyl-1,1′-biphenyl (BCMBP) and dichloromethylanthracene (BCMA), which are used to synthesize homopolymers and copolymers. Among them, BCMBP/p-DCX copolymer has the highest hydrogen storage capacity in porous polymers, which is 1.83 wt % (77K, 0.1 MPa) and 3.68 wt % (77K, 1.5 MPa). Hypercrosslinked polymers have been reported to be used in hydrogen storage due to their rich micropores and ultramicropores. Hypercrosslinked polystyrene and polyaniline also have large hydrogen storage capacity [29]. The optimal pore size for hyper-crosslinked polymers (HCPs) that enables hydrogen storage applications depends on several factors, including the type of HCP used and the conditions under which hydrogen storage is to be achieved. In general, for hydrogen storage applications, the optimal pore size of HCPs is in the range of 0.5–1.5 nm. This is because hydrogen molecules are small and require small pore sizes to be adsorbed effectively. Additionally, the pore size should be optimized to achieve a balance between high surface area and high porosity, which are critical factors in enhancing hydrogen storage capacity.

### 3.2 Polymers of intrinsic microporosity

**Figure 15.**
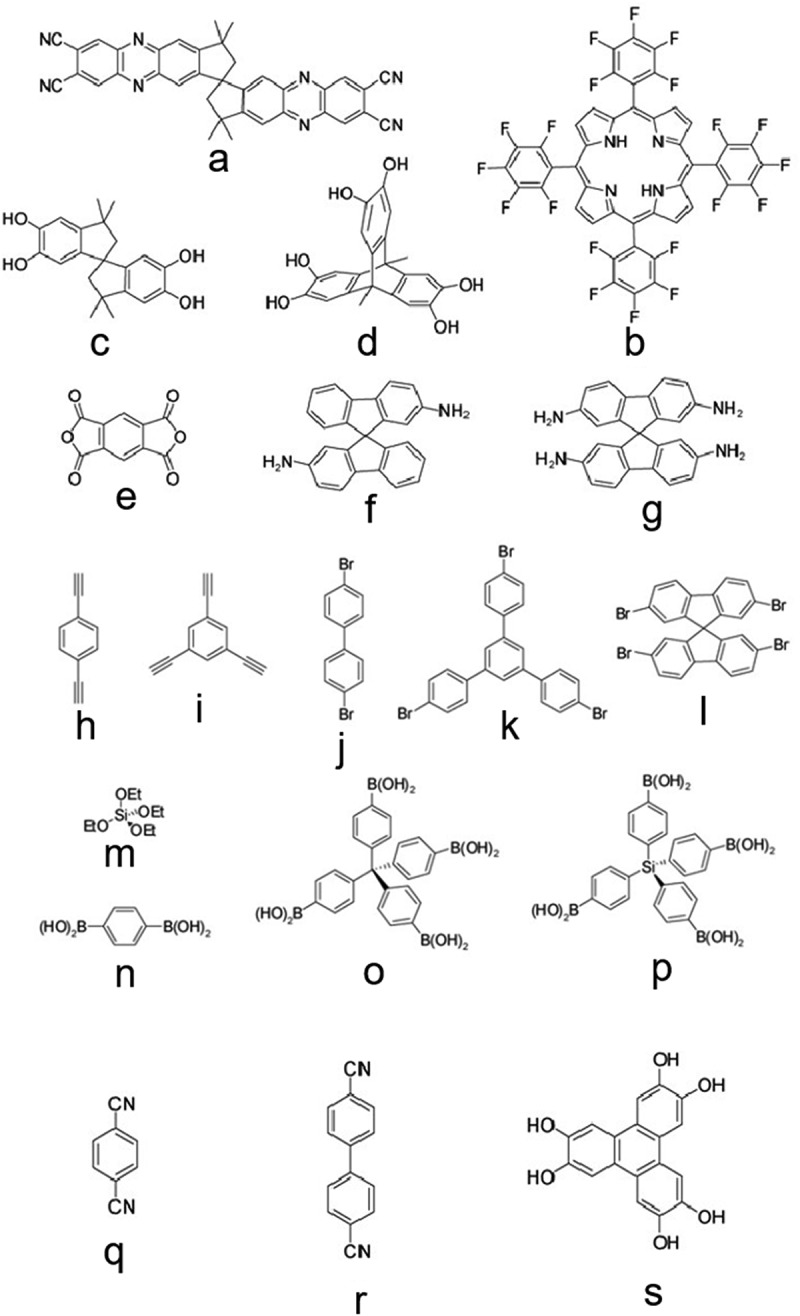
Monomers 1–19 to prepare microporous organic polymers.

**Figure 16.**
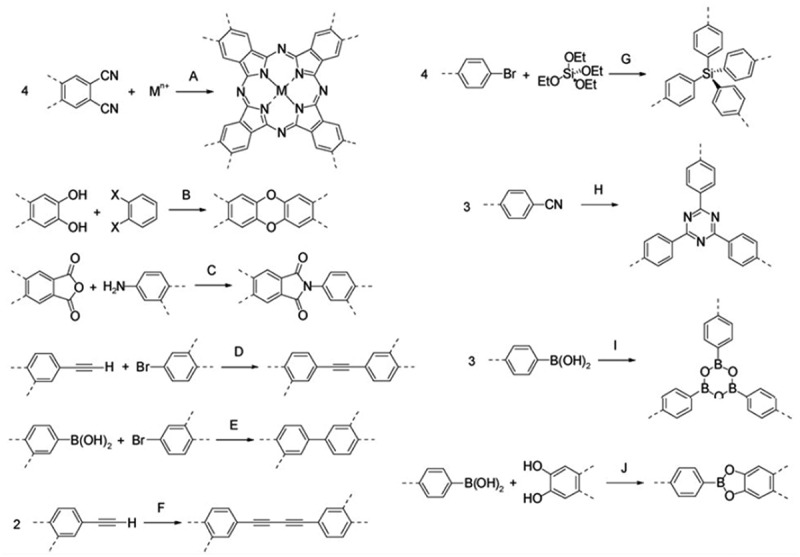
Reaction A-J used to prepare microporous organic polymers.

In general, macromolecular chains are able to maximize intermolecular interactions by bending and twisting to make it possible to stack efficiently in space. However, it has long been recognized that some polymers possess a large amount of void space, defined as free volume. If these free volumes can be interconnected in some way, it is conceivable that such polymers can exhibit some microporous properties even if they do not have a network structure. This is the reason why some soluble polymers still have a large SSAs [30]. Above the glass transition temperature (T_g_) of the polymer, there will exist a rubbery state, which the chains of the polymer can move freely to a greater extent. There will be a relatively large amount of free volume. After the temperature is cooled below T_g_, the amount of free volume will decrease, and there will be some dead space in the polymer skeleton. The polymer then behaves more like hard glass. For most polymers, a very small amount of free volume is present in the glassy state. However, for some polymers with a hard structure, a larger amount of free volume (up to 20%) is retained during cooling or rapid solvent removal [30]. PIMs are a special class of polymers that obtain microporosity by their own rigidity and the non-planar structure of the molecules. They usually have a rigid, non-planar distorted spatial configuration and can achieve specific surface areas of 500 ~ 1065 m^2^g^−1^. There is a growing body of work on PIMs, and the authors Budd, McKeown and Thomas and their collaborators have done some pioneering work on PIMs. The first example of PIMs is a polymer of phthalocyanine or porphyrins connected by twisted spirobindene centres [31,32], with the SSAs of 895 m^2^g^−1^ based on the phthalocyanine network structure (Monomer 1 (Figure 15) and the template metal ion Mn+, Reaction A (Figure 16)) [31] and the SSAs of 910 m^2^g^−1^ for the porphyrin network structure (Monomers 2 and 3 (Figure 15), Reaction B (Figure 16)) [32]. Some previously prepared simple phthalocyanine polymers, however, have a tendency for their macrocycles to pile up into columns due to the presence of strong non-covalent interactions (mainly π-πinteractions), eventually leading to the absence of a porous structure [33]. Therefore, the use of such highly rigid and non-planar linking groups between the macrocycles of two phthalocyanines effectively prevents the dense stacking of polymer chains and the free rotation between chains, thus maintaining the microporosity of the structure. A readily available monomer that satisfies this condition is 5,5′,6,6′-tetrahydroxy-3,3,3′,3′-tetramethyl-1,1′-spiro-bisindene (Monomer 3). The spiro centre (two rings sharing a single carbon atom) within this molecule provides a non-planar, twisted ring structure. Subsequently, a range of PIMs (PIMs-1, PIMs-7, etc.) were prepared, which were all linked by a wide variety of rigid molecules and non-linear groups, and the SSAs of the materials varied from 500 to over 1000 m^2^g^−1^. For example, a polymerisation reaction using a derivative of hexahydroxytriptene (Monomer 4, Figure 15) as the central molecule to form a dioxane ring (Reaction B, Figure 16) gave some non-planar polymers with a trapezoidal structure [16], in which the maximum SSAs and hydrogen storage capacity achieved were 1416 m^2^g^−1^ and 1.68 wt%, respectively (77 K, 0.1 MPa). These are prepared by a number of kinetically controlled irreversible reactions and the resulting materials are therefore amorphous. Although PIMs are amorphous, they still exhibit high SSAs and the pore size distribution is in the range of micro- and ultra-micro-pores. Often they have a wide pore size distribution that is not easily controlled and regulated. Depending on the monomer used, some PIMs can form infinite network structures (using monomers with bifunctional groups), while others cannot (using trifunctional groups or more), but they can be soluble in certain solvents. In this way the rigid and twisted groups inside the polymer prevent effective stacking between the chains and form a large number of micropores. This solubility, which facilitates the preparation of gas separation membranes by spin coating [34–36], is a property that cannot be matched by other microporous polymers.

Budd and Weber et al. [35,37] used nucleophilic substitution reactions of aromatic rings to form linkers of dioxane to prepare microporous polymers in earlier years. This approach was recently further extended by Thomas et al. who used derivatives of 9,9′-spirofluorene to prepare polyamide and polyimide PIMs. For example, the reaction of 2,2′,7,7′-diamino-9,9’-spirofluorene with homophthalic dianhydride (Monomers 6 and 5 (Figure 15), Reaction C (Figure 16)) gave a soluble polymer with the SSAs of 551 m^2^g^−1^ [37]. This is comparable to the previously reported surface area of PIMs. However, it was found experimentally that the surface area of the material was dependent on the solvent used for the precipitation. Subsequently, the reaction using the monomer 2,2′,7,7′-tetraamino-9,9’-spirofluorene (Monomer 7, Figure 15) gave a network structure of polyimide with the SSAs of 982 m^2^g^−1^ [38]. Due to the large free volume of this type of polymers, it is mostly used in non-homogeneous catalysis and gas separation membrane preparation. Hasell et al. [39] loaded palladium nanoparticles into the pores of polymers and formed hybrid materials with high catalytic activity for both Suzuki coupling and Knoevenagel condensation reactions. Some of the polymers have a certain solubility in polar solvents, and gas separation membranes can be prepared by spin-coating method, which has high selectivity for CO_2_/H_2_, H_2_/N_2_, and H_2_/CH_4_ [40].

### 3.3 Conjugated microporous polymers

CMPs have great potential for research due to their electronic and electrofluorescent properties. Most of the conjugated systems have inherently more rigid structures, and these polymers have permanent microporous structures, but little research has been done on them. Moreover, the thermal stability of CMPs is an important factor to consider for their practical applications, as it can affect their structural integrity and performance at high temperatures. In general, CMPs exhibit good thermal stability due to the presence of strong covalent bonds in their polymer backbone, which can withstand high temperatures without significant degradation. Many CMPs have been reported to exhibit thermal stability up to temperatures of 300–500 °C or higher, making them promising materials for high-temperature applications [41].

In 2007, Jiang et al. [41] reported for the first time this type of polymer-polyacetylene aryl compounds (PAEs), but due to the low SSAs and pore volume of PAE polymers, the maximum SSAs and pore volume obtained for the polymers were only 834 m^2^g^−1^ and 0.33 cm^3^g^−1^. The thermodynamic behavior of gas adsorption is very similar to that of covalent-organic frameworks (COFs), but usually COFs have a crystalline structure while CMPs have an amorphous structure. Five PAECMPs were prepared by transition metal-catalyzed Sonogashira-Hagihara cross-coupling reactions (Monomers 8 and 11 (Figure 15), Reaction D (Figure 16)) [41,42]. For the first time, the average pore size, pore volume and BET specific surface area of PAEs were adjusted by varying the length of the monomers in amorphous microporous organic polymer [21]. Similarly, Weber et al. [42] prepared spirofluorene-based polyphenylene compounds (Monomers 12 and 14 (Figure 15), Reaction E (Figure 16) and polyphenylene acetylene-based compounds by Suzuki-Miyaura cross-coupling reaction. Such compounds have a moderate BET surface area of 210–522 m^2^g^−1^. Some hysteresis loops can be seen in the adsorption isotherms of such compounds, indicating some solvation properties of the backbone structure. Both types of reactions above were prepared by cross-coupling reactions, and similar conjugated microporous structures can be obtained by their own coupling reactions, such as polyphenylbutadiene compounds (PPB) (Monomer 9 (Figure 15), Reaction F (Figure 16)) [43]. Such substances also have high SSAs, which can exceed 800 m^2^g^−1^. Due to the relatively long monomers, the presence of some mesoporous phases can be seen both from adsorption isotherms and electron microscopy photographs. The three types of CMPs mentioned above are all composed of carbon and hydrogen elements. Recently, a microporous polysilane-based polymer (Monomers 13 and 10 (Figure 15), Reaction G (Figure 16)) was reported [44] with a surface area up to 1046 m^2^g^−1^. These polymers were named ‘Element-Organic Frameworks (EOFs)’ and were prepared by organolithium reagents, which was similar to the synthesis of hypercrosslinked polytriphenylmethanol. Due to the presence of silicon elements within the polymer, it is very hydrophilic and can adsorb large amounts of water vapor [44].

Poly(p-phenylene propylene vinylidene) (PPV) is one of the most studied and promising photovoltaic materials due to its excellent photovoltaic properties. Dawson et al. [45] prepared PPV-like polymers by Gilch coupling reaction of 1,2,4,5-tetrakis(bromomethylene)benzene (TBMB) by a one-pot method, and such materials have certain mesoporous properties with the SSAs of 761 m^2^g^−1^. In the process of preparing hypercrosslinked polyaniline, a very unique method for preparing CMPs is used: it is prepared by the reaction of linear polyaniline (or its sodium salt) with a cross-linking agent (such as diiodomethane) (very similar to the preparation of HCPs) [46]. The BET surface area of the resulting hypercrosslinked polyaniline is 632 m^2^g^−1^. The adsorption isotherm is a typical IV type, indicating that there are some mesoporous properties in the material, which is further confirmed by electron microscopy. Covalent Triazine-based Framework (CTF), the only class of CMPs with a crystalline structure until now, is achieved by the trimerization reaction of cyanoacids to generate triazine rings under ionothermic conditions [47]. Using 1,4-dicyanobenzene (Monomer 17 (Figure 15), Reaction H (Figure 16)) as monomer, the BET surface area of CTF-1 can reach 791 m^2^g^−1^. If dicyanobiphenyl (DCBP) (Monomer 18, Figure 15) and a larger amount of ZnCl_2_ are applied, higher SSAs polymers can be obtained, up to 2475 m^2^g^−1^ [47]. The molten ZnCl_2_ acts as both a medium and a catalyst for the reaction and most likely also acts as a microporous template. CMPs have a larger system and can be expected to have a greater application in organic optoelectronic devices. The conjugated polymers prepared based on spirofluorene units with strong blue light emission in the solid state demonstrated their potential utilization in organic light-emitting diodes [48].

### 3.4 Covalent-organic frameworks

Among the three major classes of microporous polymers described above, all are amorphous structures except for CTFs, which have a crystalline structure. Some typical reactions for the formation of COFs are reversible and thermodynamically controlled. It can be clearly distinguished from other types of microporous solids because of the high orderliness of its crystalline structure. It is due to this orderliness that some properties of COFs, such as pore size and pore size distribution, are more homogeneous and easily controlled artificially. Its crystalline structure can be characterized by X-ray diffraction. The first reported COFs were obtained from the self-condensation of 1,4-benzenediboronic acid (Monomer 14 (Figure 15), Reaction I (Figure 16)) and the condensation reaction of 1,4-benzenediboronic acid and hexahydroxybenzophenanthrene (Monomers 14 and 19 (Figure 15), Reaction J (Figure 16)) [49]. The final SSAs of this skeletal structure were 711 m^2^g^−1^ and 1590 m^2^g^−1^, respectively, and the calculated pore size distribution is also well matched with the proposed structure. Subsequently, El-Kaderi et al. [50] successfully constructed a 3DCOFs for the first time using a molecular building block of tetrahedra. The monomers were tetraphenylmethane tetraboronic acid (TBPM) (Monomer 15, Figure 1) and tetraphenylsilane tetraboronic acid (TBPS) (Monomer 16, Figure 15), which were self-condensed or condensed with hexahydroxybenzophenanthrene (Monomer 19, Figure 15) to obtain COF-102, COF-103, COF-105, and COF-108, respectively. Among them, COF-102 has the highest SSAs of 4210 m^2^g^−1^ and COF-108 has the lowest density of 0.17 gcm^−3^. Later, Tilford et al. [51] adjusted the pore size by changing the size of the substituent on the aromatic ring, and found this series of COFs had the same topological network structure, indicating that the size of the substituent would not have much effect on the structure of the material.

Jiang’s group obtained TP-COF, PPy-COF using self-condensation of pyrene diboronic acid or co-condensation of pyrene diboronic acid with hexahydroxybenzophenanthrene, respectively, to study their applications in photovoltaics [52]. The materials have the properties of p-type semiconductors, and in particular, PPy-COF exhibits effective photoconductive properties and can respond quickly to light radiation. With the increasing energy and environmental requirements, microporous organic polymers show high utilization value with their unique advantages in non-homogeneous catalysis, adsorption, separation and gas storage. Therefore, the research and understanding of such novel microporous materials have been gradually increased. It remains a challenging task to efficiently assemble microporous organic polymers by building blocks of organic small molecules. The use of the diversity of organic molecules and the complexity of organic reactions can greatly expand the research in this field. It is desirable to prepare microporous organic polymers with higher SSAs and more topological structures to meet the needs in the energy and environmental fields.

## 4. Potential application and positions

Carbazole is a very important nitrogen-containing aromatic heterocycle with a special rigid thick ring structure, and its derivatives exhibit many unique photoelectric properties and biological activities. The structure of carbazole compounds has the following characteristics: (1) the carbazole ring is easy to form relatively stable positive ions; (2) the molecule has a large conjugation system and strong intramolecular electron transfer; (3) generally has high thermal and photochemical stability; (4) the carbazole ring is easy to make structural modifications to introduce a variety of functional groups; (5) the carbazole itself is one of the coal tar products, and raw materials are easily available. The special structure of carbazole has led to a wide range of potential applications of its derivatives in areas such as optoelectronic materials, dyes, pharmaceuticals, supramolecular recognition, etc., which have been extensively studied and developed in recent years. In the field of optoelectronic materials, carbazole derivatives like small molecule materials 4,4’-N,N’-dicarbazole biphenyl (CBP) and polymer materials polyvinyl carbazole (PVK) are the most widely studied and effective materials as organic optoelectronic materials. With the rapid development of solar cell devices, the development of organic photosensitive dyes has received increasing attention. Carbazoles have been widely used for the preparation of organic photosensitive dyes because of their good hole transport properties and high energy gap [15,16]. In the field of medicine, more than 60 carbazole alkaloids with biological activity have been isolated from Phellodendron spp. Among them, indole and carbazole alkaloids have attracted much attention from chemists and pharmacologists because of their excellent biological activity. These compounds have good inhibitory effects on protein kinase C and topoisomerase I. With the gradual discovery that carbazole analogues exhibit a broad spectrum of biological activities, the research on synthetic carbazole-like drugs has recently become more and more active. The rapid development of supramolecular chemistry has extended to the fields of supramolecular materials and supramolecular drugs [17,18], and the research on artificial receptors and molecular probes constructed on the basis of rigid skeletal structures of carbazole ring macroconjugation systems with excellent supramolecular identification properties has been increasing.

### 4.1 Optoelectronic materials field

Research on organic light-emitting materials emerged in the 1970s. In recent years, the research of organic photoelectric materials is very active. Organic molecular systems with good photostability, wide spectral absorption range, and high photoelectric conversion efficiency have received great attention from researchers. The special thick ring structure and large conjugated π-electron system of carbazole compounds give them strong optoelectronic properties, which has led to extensive research on these compounds as organic optoelectronic materials. Until now, the research on the application of carbazole compounds in the field of optoelectronic materials has involved organic photoelectric molecular devices, electronic photographic materials, solar cell materials, color liquid crystal materials and organic optical information recording materials and other frontier science and technology fields, showing a broad application prospect. Carbazole-based optoelectronic materials can be divided into organic electroluminescent materials, organic photorefractive materials and solar cell materials, etc [18,19]. In addition, many CMPs exhibit good electrochemical stability due to the presence of strong covalent bonds in their polymer backbone. For example, in a study reported by Zhang et al. in 2017, a nitrogen-rich CMP was synthesized by the polymerization of 2,3,6,7,10,11-hexaaminotriphenylene with 1,3,5-triformylbenzene. The resulting CMP showed excellent electrochemical stability up to 3.5 V vs. Ag/AgCl, as demonstrated by cyclic voltammetry [17,18]. Similarly, in a study reported by Liu et al. in 2018, a sulfur-containing CMP was synthesized by the polymerization of a thiophene-based monomer. The resulting CMP showed good electrochemical stability up to 2.8 V vs. Ag/Ag+ in acetonitrile, as demonstrated by cyclic voltammetry [18].

### 4.2 Pharmaceutical field

Carbazole and its derivatives show many important biological activities [19,21], and therefore the research and development of carbazole compounds in the pharmaceutical field is of great interest. For example, carbazole itself can be used as a stabilizer for insecticides and plant growth regulator, both chlorinated and nitro derivatives of carbazole can be used in the synthesis of insecticides, and 3-acetylamino carbazole, bromocarbazole, N-methyl carbazole, 4-hydroxy carbazole and tetrahydrocarbazone are all important pharmaceutical intermediates. The breakthrough of carbazole and its derivatives in the field of medicine started with the isolation of a class of carbazole alkaloids with a wide range of biological activities from Phellodendron spp. Carbazole alkaloids are mainly found in natural plants such as Clausena, Murraya and Glycosmis, and have been widely concerned for their pharmacological activities such as antitumor, antimicrobial, antihistamine, antioxidant and anti-inflammatory activities.

### 4.3 Supramolecular identification field

Supramolecular chemistry is a thriving interdisciplinary field with research areas involving molecular identification, molecular catalysis, molecular self-assembly, and supramolecular drugs [17,18]. Molecular recognition, the process by which a subject substance selectively binds to an object substance and produces a specific function, is an important research element in supramolecular chemistry. Fluorescence/phosphorescence detection methods have the advantages of simplicity, high sensitivity, good selectivity, fast response time, easy implementation of in situ monitoring (e.g., fluorescence imaging techniques), and the possibility of telemetry using optical fibers. Therefore, the study of modifying fluorophores on traditional host molecules to construct new supramolecular fluorescence/phosphorescence sensors for the recognition of guest molecules/ions has been quite important, and fluorescent compounds with conjugated structures and intramolecular charge transfer are among the most widely studied compounds. Because of the good delocalization of π-electrons of these compounds, the overall molecular excitation easily occurs when illuminated, which causes charge transfer and electron density redistribution within the molecule, and thus has good photoelectric properties. Carbazole and its derivatives are among the more widely studied organic fluorescent substances because of their large intramolecular conjugation system and strong intramolecular electron transfer [22,23]. The fluorescent properties of carbazole derivatives can vary depending on the nature of the substituents on the carbazole core. The substituents can be either electron-donating or electron-withdrawing, and they can affect the electron density of the carbazole core, which in turn affects its electronic and optical properties. For example, carbazole derivatives with electron-donating substituents, such as alkyl or aryl groups, typically exhibit higher fluorescence quantum yields and longer fluorescence lifetimes compared to carbazole derivatives with electron-withdrawing substituents, such as nitro or cyano groups. This is because electron-donating groups increase the electron density on the carbazole core, which enhances its ability to absorb and emit light [22]. On the other hand, carbazole derivatives with electron-withdrawing substituents typically exhibit bathochromic shifts in their absorption and emission spectra, meaning that their absorption and emission wavelengths are shifted to longer wavelengths. This is because electron-withdrawing groups decrease the electron density on the carbazole core, which affects its electronic and optical properties [23]. It is believed that with the development of supramolecular chemistry, especially the close integration of supramolecular chemistry research with contemporary high-tech fields, the research of carbazole compounds in the field of supramolecular chemistry will certainly extend to several hot research directions such as molecular catalysis, molecular and supramolecular devices, molecular self-assembly, simulated enzyme models and supramolecular drugs [15,16], in addition to molecular recognition.

### 4.4 Other fields

The reaction of carbazole as raw material with a variety of compounds can obtain special plastics and rubbers, such as carbazole with phenol and formaldehyde can be condensed to phenolic linear varnishes, amino light ride, etc. Dyes containing carbazole aromatic heterocycles are also widely used in the field of dyestuffs because of their bright colors, strong coloring power, and resistance to fading [24]. Many of them also have strong acid and alkali resistance and are commonly used in dyeing and printing of polyester, blended fibers, fabrics and in the plastic industry.

Carbazole condensation with phenol and formaldehyde can prepare excellent performance of concrete water reducing agent, and with ethylene oxide can synthesize special surfactants, and also can synthesize stabilizers for lubricants and heat transfer oils. Carbazole compounds are also used in the preparation of explosives, rubber antioxidants, etc., and can be used as photosensitive initiators to control the photopolymerization reaction (e.g., benzyl 9 H-carbazole-9-carbonyldisulfate) [25]. In addition, many new applications of carbazole are being developed and research on their industrial applications is active.

## 5. Conclusion

In conclusion, the comprehensive study on carbazole derivatives presented in this review highlights the significant progress made in the synthesis and application of these compounds in various fields such as optoelectronics, sensing, and catalysis. The versatility of carbazole derivatives, coupled with their unique electronic and optical properties, makes them promising candidates for a wide range of applications.

The review has also identified several areas for future research, such as the development of new synthetic routes, investigation of structure-property relationships, and exploration of new applications. The research community’s continued efforts in these areas will undoubtedly lead to new and exciting discoveries and further cement carbazole derivatives as promising materials for versatile applications.

Overall, this review underscores the significance of carbazole derivatives as a class of compounds with tremendous potential for diverse applications. We hope that this review will motivate and inspire researchers to explore new avenues for the synthesis and application of carbazole derivatives, thus contributing to the development of innovative materials with novel properties and functionalities.
